# Identify potential driver genes for PAX-FOXO1 fusion-negative rhabdomyosarcoma through frequent gene co-expression network mining

**DOI:** 10.3389/fonc.2023.1080989

**Published:** 2023-01-30

**Authors:** Xiaohui Zhan, Yusong Liu, Asha Jacob Jannu, Shaoyang Huang, Bo Ye, Wei Wei, Pankita H. Pandya, Xiufen Ye, Karen E. Pollok, Jamie L. Renbarger, Kun Huang, Jie Zhang

**Affiliations:** ^1^ Department of Bioinformatics, School of Basic Medicine, Chongqing Medical University, Chongqing, China; ^2^ College of Intelligent Systems Science and Engineering, Harbin Engineering University, Harbin, China; ^3^ Department of Biostatistics and Health Data Science, Indiana University, School of Medicine, Indianapolis, IN, United States; ^4^ Carmel High School, Indianapolis, IN, United States; ^5^ Department of Pediatrics, Indiana University, School of Medicine, Indianapolis, IN, United States; ^6^ Department of Medical and Molecular Genetics, Indiana University, School of Medicine, Indianapolis, IN, United States

**Keywords:** fusion-negative RMS (FN-RMS), fusion-positive RMS (FP-RMS), frequent co-expression network (fGCN), copy number alteration, upstream regulator

## Abstract

**Background:**

Rhabdomyosarcoma (RMS) is a soft tissue sarcoma usually originated from skeletal muscle. Currently, RMS classification based on PAX–FOXO1 fusion is widely adopted. However, compared to relatively clear understanding of the tumorigenesis in the fusion-positive RMS, little is known for that in fusion-negative RMS (FN-RMS).

**Methods:**

We explored the molecular mechanisms and the driver genes of FN-RMS through frequent gene co-expression network mining (fGCN), differential copy number (CN) and differential expression analyses on multiple RMS transcriptomic datasets.

**Results:**

We obtained 50 fGCN modules, among which five are differentially expressed between different fusion status. A closer look showed 23% of Module 2 genes are concentrated on several cytobands of chromosome 8. Upstream regulators such as MYC, YAP1, TWIST1 were identified for the fGCN modules. Using in a separate dataset we confirmed that, comparing to FP-RMS, 59 Module 2 genes show consistent CN amplification and mRNA overexpression, among which 28 are on the identified chr8 cytobands. Such CN amplification and nearby MYC (also resides on one of the above cytobands) and other upstream regulators (YAP1, TWIST1) may work together to drive FN-RMS tumorigenesis and progression. Up to 43.1% downstream targets of Yap1 and 45.8% of the targets of Myc are differentially expressed in FN-RMS vs. normal comparisons, which also confirmed the driving force of these regulators.

**Discussion:**

We discovered that copy number amplification of specific cytobands on chr8 and the upstream regulators MYC, YAP1 and TWIST1 work together to affect the downstream gene co-expression and promote FN-RMS tumorigenesis and progression. Our findings provide new insights for FN-RMS tumorigenesis and offer promising targets for precision therapy. Experimental investigation about the functions of identified potential drivers in FN-RMS are in progress.

## Graphical Abstract

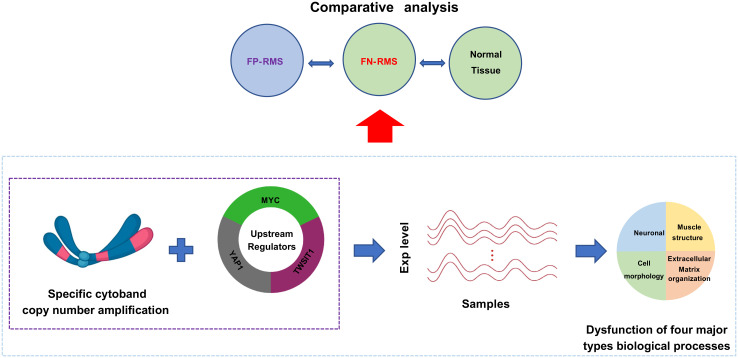

## 1 Introduction

Rhabdomyosarcoma (RMS) is one of the most common soft tissue sarcomas in children. It accounts for about 5% of all childhood tumors and represents about 50% of pediatric soft tissue sarcomas cases (1, 2). RMS usually originates and develops from skeletal (striated) muscle cells (3). Traditionally, RMS contains two major subtypes: embryonal RMS (ERMS; accounts for ~60% of RMS cases) and alveolar RMS (ARMS; ~20% of RMS cases); each presents distinct histologic features, molecular alterations, and clinical outcome (2, 3). At the molecular level, ARMS could be further divided into two subtypes according to the presence or absence of the PAX3 or PAX7–FOXO1 gene fusion (1, 4), which refers to the chromosomal translocations t(2;13)(q35;q14) and t(1;13)(p36;q14). A part of *PAX3* (on chromosome 2) or *PAX7* (on chromosome 1) is fused to *FOXO1* (on chromosome 13) gene (4). ARMS with PAX3/PAX7-FOXO1(together called PAX-FOXO1) gene fusion represents about 80% of ARMS cases (1, 4, 5), and the ones without fusion gene accounts for the rest 20%, which exhibit similar molecular patterns and clinical behaviors to ERMS. This indicates that the fusion status can better classify patient outcomes than the histologic subtype (1, 4). Thus, this molecular level classification with respect to PAX–FOXO1 fusion status has been gradually adopted. In this work, we refer to PAX-FOXO1 fusion-positive cases as FP-RMS and the others as FN-RMS. FP-RMS contains relatively low-overall somatic mutation burden (1); therefore, a number of studies have been carried out to investigate whether PAX-FOXO1 gene fusion functions as the driver for the tumorigenesis and development of FP-RMS. Ren et al. (6) confirmed that PAX-FOXO1 fusion genes commit mesenchymal stem cells to a myogenic lineage by inhibiting terminal differentiation and contributing to ARMS formation. Another study showed that PAX-FOXO1 fusion activates transcription factor (TF) *MYOD* and myogenin and transforms mesenchymal progenitor cells to the skeletal muscle lineage, leading to malignant formation resembling ARMS (6). Rarer gene fusion of *PAX3* to *NCOA1* (7) or *NCOA2* (8) have been reported, but because they are much rarer and *NCOA1/2* functions similarly as *FOXO1*, those fusions can be treated as special cases of PAX-FP RMS.

However, compared with relatively clear understanding of tumorigenesis and driving force in FP-RMS, the potential driver genes of the more heterogeneous FN-RMS, which represent more than 80% of the RMS cases, are still unclear. Previously, several SNVs for FN-RMS have been identified such as RAS as a potential driver gene. RAS mutations has been detected in 5–30% of all RMS patients (9). However, they are only significantly associated with a subset of ERMS patients (75% of high-risk and 45% of intermediate-risk cases had RAS mutations, whereas low-risk ERMS had no mutations) (10), and not with all of the FN-RMS patients. The understanding about the mechanisms of FN-RMS as a whole is still incomplete, and further efforts to look for other potential driver genes of this group are still needed. Moreover, while FN-RMS harbor many more SNVs, these SNVs are barely linked to clinic due to limited recurrent SNVs across RMS specimens, lack of ability to target a cancer-driving mutation, and incorrect assumptions about the relevance of individual aberrations (11–15). Accumulated studies have pointed out that DNA copy number variations (CNVs) are common and more highly recurrent in FN RMS (15, 16). In previous genomic analysis, ERMS (all considered as FN-RMS) demonstrates a high frequency of LOH on chromosomes 11p, 11q, and 16q (1) and tends to have more CNV than ARMS (1). However, a more systematic comparative analysis of FN- *versus* FP-RMS is still needed to pinpoint the driving force behind FN-RMS tumorigenesis and development, as well as to identify promising therapeutic targets for FN-RMS.

Gene co-expression network mining identifies groups of genes possessing highly correlated/anti-correlated expression profiles across samples or disease conditions. These so-called gene co-expression network modules (GCN modules) are generally enriched in specific pathways or biological functions. Some module’s co-expression is the result of common upstream regulators such as TFs. Therefore, detecting GCN modules specific to a disease condition can lead to quick identification of potential driving regulators. Alternatively, module co-expression can also result from the chromosomal CNVs. Genes in such modules reside on the same fragment of a chromosome, and the CN changes cause their expression levels to vary synchronously. Therefore, GCN mining can also serve as a powerful tool to identify both functional CNVs and driving regulators. This approach has been successfully applied to multiple adult cancer studies by our group on colorectal, breast, lung, and kidney cancers to identify potential driver genes and CN changes (17–21).

In this study, we aim to identify the driving mechanism of FN-RMS through the coexpression network mining, which can quickly pinpoint down the potential CNV changes as well as the pathway-level changes in the FN-RMS including ERMS. We systematically investigated the molecular mechanisms and the potential upstream regulators of FN-RMS with the gene co-expression network mining approach. First, we applied our previously developed frequent co-expression network (fGCN) mining algorithm to identify frequent (or consensus) co-expression network modules (called fGCN modules) across multiple RMS gene expression datasets and obtained 50 fGCN modules. Second, we explored the distinctive co-expressed modules between FP- and FN-RMS, and five modules (Modules 2, 18, 34, 41, and 46) enriched with genes differentially expressed between the two groups were detected. For these five modules, we observed that four major types of biological processes (namely, extracellular matrix organization, cell morphology, neuron development, and muscle structure related functions) were highly enriched. Specifically, 117 of 504 genes in Module 2 are concentrated on several cytobands on chromosome 8; Module-enriched upstream regulators such as Myc, Yap1, and Twist1 presented distinctive expression patterns between two groups. Moreover, we also found that up to 24% of the Myc target genes and 27% of the Yap1 (through TEA/ATTS activators) target genes are differentially expressed in the FN *versus* FP-RMS in three datasets. More importantly, up to 45.8% of Myc target genes and up to 43.1% of Yap1/TEA/ATTS targets are differentially expressed in FN-RMS *versus* normal comparisons (**Table 5**). These observations promoted us to hypothesize that the CN changes on these enriched cytobands and the upstream regulators work together to generate the co-expression pattern in FN-RMS and contribute to tumor development. To verify our hypothesis, we performed differential CN and gene expression analyses on a separate RMS dataset from St. Jude Children’s hospital. We observed the consistent CN amplification and overexpression patterns comparing FN-RMS with FP-RMS at both discovery datasets and St. Jude validation dataset. Meanwhile, we also detected the same patterns for three transcriptional regulators (*MYC*, *YAP1*, and *TWIST1*). Notably, *MYC* is located on chr8q24, one of the identified cytobands by fGCN analysis, whereas *YAP1* and *TWIST1* were previously reported RMS oncogenes or muscle development genes.

In summary, we identified specific cytobands CN amplification on chromosome 8 as well as the enriched upstream transcriptional regulators through GCN mining and validated the findings through genomic and transcriptomic analysis. We believe that those upregulated gene expression through CN amplification combined with those transcriptional regulators work together to induce the gene co-expression and subsequently may drive the FN-RMS tumorigenesis and development. Our findings not only provide new insights for tumorigenesis and development of fusion-negative RMS but also offer promising directions for FN-RMS driving force study and new potential targets for precision therapy. Experimental validations work is currently going to further validate the roles of the potential driver genes in the FN-RMS samples and cell lines.

## 2 Materials and methods

### 2.1 Data source

Five RMS datasets including GSE66533 (22), GSE108022 (23), GSE114621 (15), GSE28511 (24), and St. Jude Children’s hospital dataset (https://www.stjude.cloud/ ) were used in our analysis. Among the five, the first three datasets (GSE66533, GSE108022, and GSE114621) served as the discovery datasets and the last two datasets (GSE28511 and St. Jude dataset) were served as the validation dataset. For the discovery datasets and GSE28511 validation dataset, both gene expression data and corresponding clinical data were obtained from the NCBI Gene Expression Omnibus Database (https://www.ncbi.nlm.nih.gov/geo/). For GSE66533 and GSE108022 data, the PAX–FOXO1 fusion status for each sample are available. In GSE108022, the three samples marked as “other fusion” types were excluded from the downstream comparative analysis and the five samples marked as “normal” were used for validation analysis. For GSE114621, only 11 samples were known for their fusion status and seven of the 11 were included in GSE108022. Therefore, GSE114621 dataset was excluded from the further comparative analysis between FP- and FN-RMS samples. For GSE28511, skeletal muscle, tumor adjacent skeletal muscle, and ERMS and ARMS samples were available. Here, the eight ERMS samples (all FN-RMS) and six normal samples (skeletal muscle and tumor adjacent skeletal muscle samples) were used for targets validation. For the St. Jude dataset, matched CN data, expression data, and clinical data were obtained. The CN data were downloaded from UCSC Xena database (http://xena.ucsc.edu ), and the gene expression data and matched clinical data were obtained from St. Jude Cloud (https://www.stjude.cloud ). Here, the 12 samples taken from initial diagnosis were chosen for analysis. The PAX–FOXO1 fusion status of each sample was manually checked using IGV browser and confirmed by Chen et al. (12). In addition, the target genes of the upstream regulators Myc and TEAD family were obtained from the TF Target Gene Database (25). More details about the samples are summarized in **Table 1**.

**Table 1:** Demographic of RMS samples and their PAX-FOXO1 fusion status in the cohorts used in the study.

Dataset		FP-RMS	FN-RMS	Others	Normal
The discovery datasets	GSE66533	33	25	0	0
	GSE108022	32	66	3	5
	GSE114621*	6	5	90	0
The verification datasets	GSE28511	10	8	0	6
	St. Jude dataset	4	8	4	0

*Some patients have missing information in these categories. Only 11 samples were provided the fusion status according to Shern et al. (2014). Among them, four in six FP-RMS and three in five FN-RMS were included in GSE108022.

### 2.2 Analysis workflow and statistical analyses

**Figure 1:**
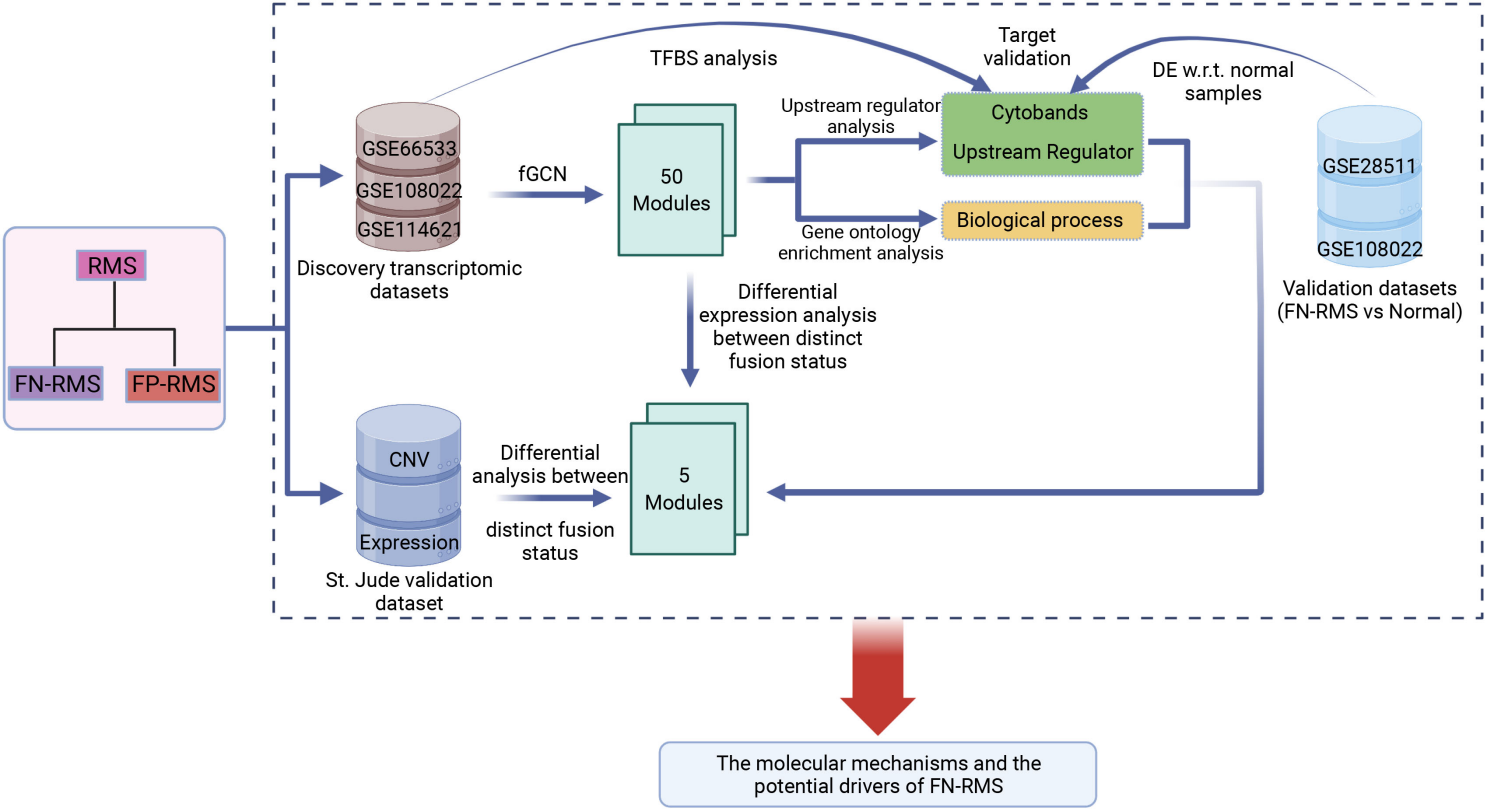
Study workflow: fGCN, frequent co-expression network minging; DE w.r.t. normal samples, Differential expression analysis with respect to normal tissue.

The main objective of this study was to investigate the molecular basis and the potential driver genes of PAX–FOXO1 fusion-negative RMS. The whole workflow is shown in **Figure 1**. First, fGCN mining was applied to three RMS gene expression datasets (GSE66533, GSE108022, and GSE114621, **Table 1**) using our developed lmQCM (local maximal quasi-clique merger) to identify frequent (consensus) GCN (fGCN) modules across the datasets. Second, the differential expression analysis was performed between different fusion status samples, and modules with significantly enriched differential co-expressed genes were further investigated. Third, Gene Ontology (GO) enrichment analysis and cytoband enrichment analysis were carried out for all the fGCN modules to obtain the associated biological processes and chromosomal regions with ToppGene (26). In addition, we also explored the potential upstream regulators for all the fGCN modules with Ingenuity Pathway Analysis (IPA) software (27). Fourth, the target genes for identified TFs were checked for their differential expression with respect to fusion status as well as between FN-RMS and normal samples. Finally, we validated our findings on both CN variation level and gene expression level using the independent validation dataset from St. Jude Children’s hospital.

### 2.3 Frequent co-expression network mining and identification of fusion-negative RMS significant modules

We applied the frequent co-expression network (fGCN) mining tool lmQCM (17) to identify frequent co-expression network modules (fGCN modules) across multiple RMS gene expression datasets (i.e., GSE66533, GSE108022, and GSE114621). This tool has been extensively used to mine tightly connected fGCN modules from multiple datasets in cancers and other diseases (17–20) and is capable of generating small and densely connected modules with highly enriched GO terms. To remove noise, genes with a value of zero in more than 50% of the samples in each dataset were excluded, and protein-coding genes were selected for future analysis. To identify the fGCN modules, we firstly calculated the Pearson correlation coefficient (PCC) between each pair of genes in each dataset separately. The gene pairs with top 5 percentile |PCC| among each dataset were chosen for subsequent analysis. Then, the frequency of gene pairs appeared in the top 5 percentile lists were computed among multiply datasets. Finally, by using the frequency as weight, we identified the fGCN modules. The parameters setting were γ = 0.7, Λ = 1.0, *t* = 1.0, β = 0.4, minimum size of cluster = 10, and adopted PCC to calculate gene-wised correlations.

To identify key modules associating with fusion status, differential expression analysis for each module gene was performed using R package Limma-voom (28). Only genes with both Benjamini–Hochberg adjusted *q*-values < 0.05 and |fold change| > 1.5 were considered as differential expressed genes (DEGs). Fisher’s exact test was further applied to identify the modules enriched with DEGs between FP-RMS and FN-RMS with the cutoff *p*-value < 0.05.

### 2.4 Cytoband and functional enrichment analysis

To identify the associated biological functions or chromosome locations of a particular fGCN module, we performed GO enrichment analysis and cytoband enrichment analysis using ToppGene (https://topgene.cchmc.org) (26) with the above fGCN module genes. Only GO terms or cytoband with false discovery rate (FDR) < 0.05 were considered to be significantly to the particular fGCN module.

### 2.5 Identification of enriched upstream transcriptional regulators for fGCN modules

To identify the potential upstream regulators of a particular module, we performed the “Core” and “Upstream Regulator” analysis with IPA (27) on identified fGCN module genes. First, we explored the potential upstream regulators for each module. Notably, we only limited to the experimentally validated interactions between upstream regulators and their target genes that have been manually curated in IPA’s Knowledge Base. Next, we further selected the potential upstream regulators for each module as follows: (1) The predicted upstream regulator itself is a transcription factor (TF); (2) The predicted upstream regulator is significantly enriched (Fisher’s exact test with *p*-value < 0.01); (3) The predicted upstream regulator affected over 10% of the examined module genes. To further investigate the transcriptional factors’ effect, target genes of Myc and Yap1 (through the interaction with TEAD family protein) were obtained from Transcription Factor Target Gene Database (http://http://tfbsdb.systemsbiology.net/) and further examined for their differential expression with respect to normal tissues in GSE108022 and GSE28511, which are the only dataset contains normal samples. The DEGs were called with the same package and thresholds as described in section 2.4.

### 2.6 Differential expression analysis for the target genes of the identified TFs

To check the effect of upstream regulators on FN-RMS, we obtained the target gene list from Transcription Factor Target Gene Database for Myc and Yap1 (through TEA/ATTS family activators). For each target to Myc or Yap1, differential expression analysis was performed to examine their molecular patterns with respect to fusion status. Furthermore, differential expression analysis between FN-RMS and normal was also carried out within GSE108022 and GSE28511 validation dataset. Moreover, the cytoband enrichment analysis for DEGs within Module 2 was performed to evaluate the influence of specific cytoband CN alteration on FN-RMS. DEGs with both Benjamini–Hochberg adjusted *q*-values < 0.05 and |fold change| >1.5 were selected.

### 2.7 Validation of the CNV and differential gene expression on St. Jude RMS samples

We examined the molecular patterns of identified five fGCN modules at both CN level and transcriptomic level in St. Jude RMS data. Particularly, we identified the modules that are enriched with differentially expressed genes and differential CNVs between different fusion status samples. In the CN level, we first performed differential CN analysis on the gene level for each module gene between FP- and FN-RMS. Wilcoxon rank-sum test method was used and *p*-values less than 0.05 was considered to be significantly different. Then, we applied Fisher’s exact test to identify fGCN modules exhibiting significant CNV difference with respect to fusion status. CN information was directly downloaded from St. Jude UCSC Xena Database. Differential CN with *p* < 0.05 were selected. Similarly, to identify the modules enriched with differential gene expression, we performed the same DEG analysis as for the two discovery datasets described in the above section. DEGs with both Benjamini–Hochberg adjusted *q*-values < 0.05 and |fold change| > 1.5 were selected. Then, Fisher’s exact test was further applied to check whether such kind of module was significantly different with respect to fusion status. *P*-values less than 0.05 were considered to be significantly different.

### 2.8 Statistical analysis software

Except where noted above, all statistical analyses were performed in R version 3.5.1.

## 3 Results

### 3.1 Identification of key modules associated with fusion status in RMS

**Figure 2:**
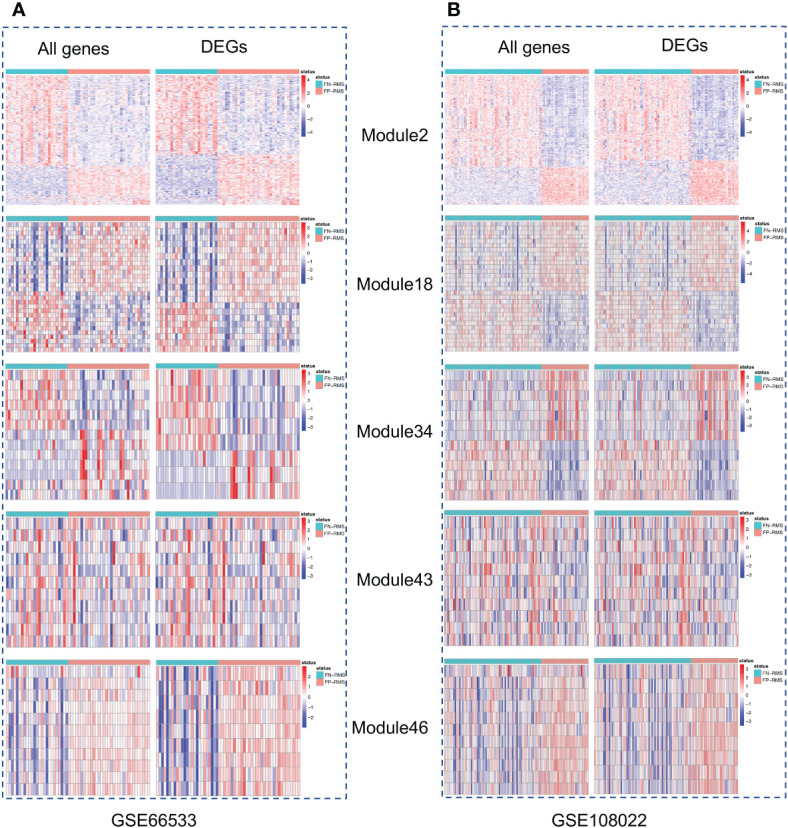
Heatmaps of the five modules significantly associated with fusion status. For each module, left is the heatmap with all module genes and right is the heatmap with differential expressed module genes. **(A)** is for GSE66533 dataset and **(B)** is for GSE108022 dataset.

Gene co-expression network mining has been proven as an effective and efficient approach to infer tumor mechanisms with transcriptomic data for potential driver genes or drug targets (17–21). To obtain robust and consensus co-expression modules in RMS, we performed frequent co-expression network (fGCN) analysis across multiple RMS gene expression datasets and 50 consensus gene expression modules were generated (**Supplementary Table S1**). Considering the distinctions exist with regard to tumor progression and prognosis between FP and FN-RMS, we focused on identify the modules enriched with genes transcriptomics different between the fusion status. Therefore, we extracted the modules enriched with differentially expressed genes using transcriptomic data from GSE66533 and GSE108022 individually (GSE114621 was omitted as the fusion status of most samples are not available). In GSE66533, six fGCN modules are enriched with DEGs between different fusion status samples (**Supplementary Table S2**), whereas in GSE108022, seven fGCN modules are enriched with such DEGs (**Supplementary Table S2**). Specifically, among them, five modules, which are fGCN Modules 2, 18, 34, 41, and 46, are shared between the two datasets. As seen from **Table 2**, the majority of the five fGCN module genes are differentially expressed between different fusion status samples in both GSE66533 dataset and GSE108022 dataset. 58.53% (295/504) genes in Module 2, 70% (21/30) genes in Module 18, 61.54% (8/13) genes in Module 34, 66.67% (8/12) genes in Module 41, and 81.82% (9/11) genes in Module 46 showed significant differential expressions between FN and FP samples in both GSE66533 and GSE108022 (**Table 2**). Consistent with this, the heatmaps of these modules also indicate such expression difference in GSE66533 and GSE108022 data (**Figure 2**). All these results suggested the five consensus modules present consistent expression within them but distinctive molecular patterns between different fusion statuses. Therefore, these five modules were further examined with GO enrichment analysis using ToppGene (26) and IPA (27).

**Table 2:** Modules associated with fusion status in both discovery datasets.

Module name	GSE66533		GSE108022		Module	
	No. DEGs	Fisher’s exact *P*-value	No. DEGs	Fisher’s exact *P*-value	Module size	No. common DEGs in both datasets
Module 2	309	2.41E-183	418	3.04E-147	504	295
Module 18	21	6.11E-15	28	1.15E-13	30	21
Module 34	8	8.46E-06	13	7.97E-08	13	8
Module 41	8	3.57E-06	12	2.81E-07	12	8
Module 46	9	4.79E-08	9	3.72E-04	11	9

DEGs, differentially expressed genes.

### 3.2 The functional enrichment of identified modules

For five consensus fGCN modules that are significantly differentially expressed between FN- and FP-RMS samples, we observed that four major types of biology processes, that is, extracellular matrix organization, cell morphology, neuron development, and muscle structure related functions are highly enriched for those modules, suggesting that they may play important roles in RMS development and progression (**Table 3** and **Supplementary Table S3**). Extracellular matrix organization (FDR = 2.05E-05), neuron development (FDR = 3.67E-05), and cell morphogenesis (FDR = 3.30E-04) related biological processes were highly enriched for Module 2 genes. Muscle structure-related biological processes were significantly enriched in Module 18 and 46 genes (FDR = 2.78E-05 and 1.35E-05, separately; **Table 3** and **Supplementary Table S3**). For GO enrichment analysis results on all the 50 modules, please see the **Supplementary Table S3**.

**Table 3:** The summary of GO-terms and upstream regulator enrichment analysis of the five fusion status associated fGCN modules.

Module	Size	Enriched cytoband	Enriched biological function/process	Enriched TFs	DE w.r.t fusion
2	504	8p24.3, 8p21.3, 8p11.2…	Extracellular matrix organization, Cell morphogenesis, neuron development	HNF4A, TP53	Yes
18	30	NA	Striated muscle contraction	MYOD1, YAP1, MYOG, KDM5A, KLF11, GATA4, MEF2C,	Yes
34	13	NA	NA		Yes
41	12	NA	Extracellular matrix organization	TP53, MYCN, TP73, HNF1B, TWIST1, IKZF1, MYC,	Yes
46	11	NA	Muscle contraction	MYOD1, SMARCA4, SRF, GATA4, MEF2C, TP53, HAND2, MYOCD, TBX5, KDM5A, RB1, CTNNB1	Yes

Sig.DE, significantly differentially expressed.

Red font indicates significantly differentially expressed.

NA indicates not significantly enriched.

### 3.3 The potential upstream regulators for the consensus fGCN modules

**Figure 3:**
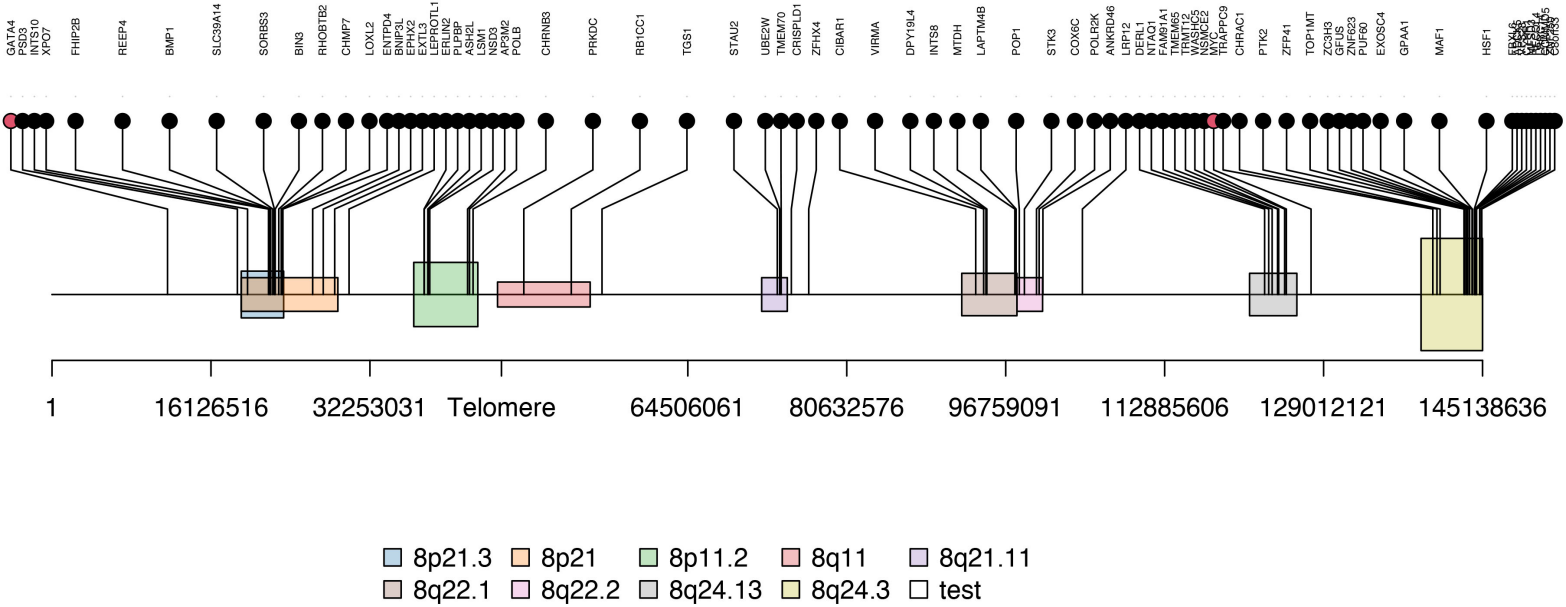
The enriched cytobands of Module 2 genes. Black dots are module 2 genes enriched on chromosome 8 and red dots are transcription factor (i.e. MYC and GATA4) located near or on the identified Module 2-enriched cytobands.

As consensus fGCN modules are commonly associated with CNVs or co-regulated by common regulators such as TFs, we first checked the chromosome co-localization of the module genes. Cytoband enrichment analysis was performed based on the module genes using ToppGene (**Supplementary Table S3**). Interestingly, 117 of 504 Module 2 genes are significantly enriched on chr8 cytobands of 8q24.3 (23 genes), 8p21.3 (12), 8p11.2 (8), 8q24.13 (7), 8q22.1 (7), and so forth (**Figure 3**).

Next, to systematically explore how regulators such as the TFs may play their roles in the consensus modules, for each module, we carried out the TF enrichment analysis using IPA Upstream Regulator Analysis (27) on the fGCN genes and the potential upstream regulators for each module were obtained (**Table 3**). We found that several consensus modules were regulated by several common upstream regulator genes *YAP1*, *KLF11*, *TWIST1*, *MYC*, *GATA4*, *MYOG*, *MYOD1*, *MYCN* and so forth (**Table 3**). Among them, most are differentially expressed between different fusion status in both GSE66533 and GSE108022. They are either previously known to be oncogenic regulators (*GATA4*, *MYC*, and *YAP1*) or myogenic or muscle differentiation factors (*GATA4*, *MYOG*, *MYOD1*, and *TWIST1*) (**Table 3**). More specifically, *YAP1*, *KLF11*, *TWIST1*, and *MYC* are upregulated, whereas *MYCN, MYOD1*, *MYOG*, and *GATA4* are downregulated in FN- *versus* FP-RMS samples (**Table 4**). *MYC* and *TWIST1* are the upstream regulators of Module 41, and *YAP1* is the upstream regulator of Module 18 (**Table 3**). Most notably, two such regulators are actually located near or on the identified Module 2–enriched cytobands (*MYC* on chr8q24.21; *GATA4* on chr8p23.1).

**Table 4:** The differential expression and copy number change for the enriched upstream regulators for the identified modules.

Gene symbol	GSE66533			GSE108022			St. Jude							
							CNV					mRNA		
	FDR	logFC FN *vs.* FP	Change FN *vs.* FP	FDR	logFC FN *vs.* FP	Change FN *vs.* FP	Pvalue	FN mean	FP mean	FN.mean- FP.mean	Change FN *vs.* FP	FDR	logFC FN *vs.* FP	Change FN *vs.* FP
**YAP1**	0.004	0.911	Up	1.00E-06	1.125	Up	0.037	0.514	0	0.514	Amp	0.011	2.912	Up
**TWIST1**	0.037	1.252	Up	0.006	1.365	Up	0.084	0.409	-0.098	0.506	n.s.	0.02	5.211	Up
**KLF11**	0.022	0.759	Up	1.03E-06	0.958	Up	0.441	0.555	0.38	0.175	n.s.	0.053	2.488	Up*
**GATA4**	3.87E-05	-1.501	Down	5.33E-29	-8.216	Down	0.017	0.783	0	0.7825	Amp	0.057	-5.181	Down*
**MYC**	0.01	1.182	Up	0.001	0.992	Up	0.0174	0.904	0	0.904	Amp	0.058	3.302	Up*
**MYCN**	8.14E-07	-1.796	Down	1.77E-11	-2.422	Down	0.733	0.563	1.135	-0.573	n.s.	0.064	-3.19	n.s.
**MYOG**	1.03E-05	-1.457	Down	2.00E-04	-1.36	Down	0.361	0.149	0	0.149	n.s.	0.460	-1.515	n.s.
**MYOD1**	0.001	-1.25	Down	2.42E-04	-1.663	Down	0.037	0.489	0	0.489	Amp	0.989	-0.026	n.s.

n.s, not significant; *above but very close to the significant p-value cutoff.

Furthermore, to confirm the upstream regulator activity changes actually caused downstream a cascade of gene expression changes, we checked the relationships between upstream regulator and their downstream targets within different fusion status. We observed that up to 24% of the *MYC* target genes and 27% of the *YAP1/TEAD* target genes (*YAP1* acts on genes through TEA/ATTS family transcription activators) are differentially expressed in the FN- *versus* FP-RMS in the discovery datasets (**Table 5**). To check whether the same trend also exists for the tumor *versus* normal sample, we examined the same set of target genes expression in FN-RMS *versus* normal tissue comparisons and found that 18.9–45.8% of the Myc target genes are differentially expressed in FN *versus* normal samples. For Yap1/TEA/ATTS family targets, the percentage of targets with altered expression is 15.2–43.1% (**Table 5**). Meanwhile, we observed that for Module 2, 25% genes in GSE28511 and 52.9% genes in GSE108022 were differential expressed when comparing FN-RMS with normal tissues (**Supplementary Figure S1**). These DEGs were also significantly enriched in several cytobands on Chr8 (**Supplementary Table S4**).

**Table 5:** Target genes differential expression in multiply datasets.

MYC target genes (Total No. 391)					
DEGs	FN *vs.* FP			FN *vs.* Normal	
	GSE66533	GSE108022	St. Jude	GSE28511	GSE108022
Total	36	94	21	74	179
Up-reg	24	58	8	45	110
Down-reg	12	36	13	29	69
YAP1/TEAD target genes (Total No. 4068)					
DEGs	FN *vs.* FP			FN *vs.* Normal	
	GSE66533	GSE108022	St. Jude	GSE28511	GSE108022
Total	457	1107	256	622	1752
Up-reg	274	684	174	283	842
Down-reg	183	423	82	339	910

Because over 23% (117 of 504) genes from Module 2 are enriched on specific cytobands, we hypothesized that the strong co-expression may not only results from common regulators but also results from the CN alteration on those cytobands.

### 3.4 CN changes play important roles in fusion-negative RMS

**Figure 4:**
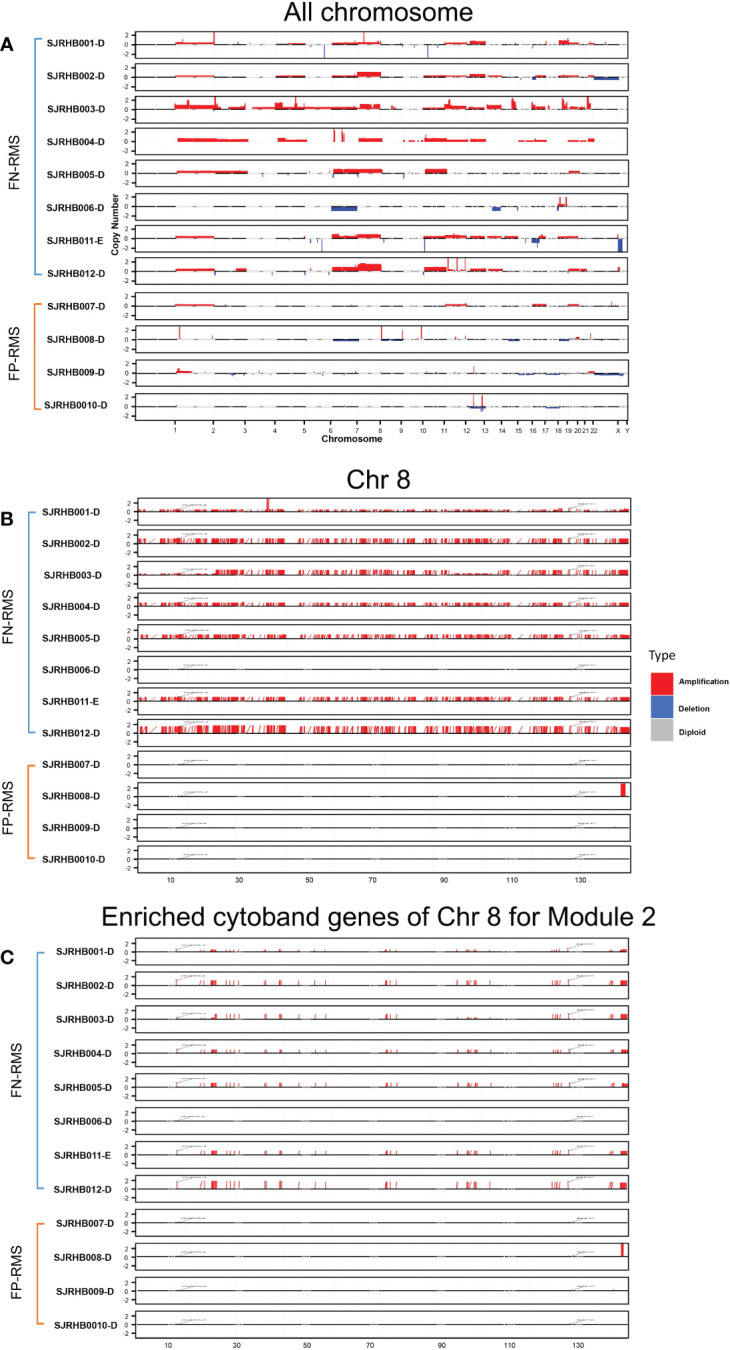
CNV distribution in FN-RMS *vs.* FP-RMS of St. Jude dataset. **(A)** is for entire genome,**(B)** is for chromosome 8, and **(C)** is for enriched cytoband genes of chr 8 for Module 2 from discovery datasets.

To test our hypothesis, we obtained CN information for a separate RMS dataset from St. Jude Children’s hospital and performed differential CN and gene expression analyses for the fusion status related modules (Modules 2, 18, 34, 41, and 46). The St. Jude data contain eight FN-RMS and four FP-RMS samples with matching RNA-seq and CN data. Overall, a visual examination of the entire genome shows that FN samples contain much more CN amplification than FP samples, especially in the chr8 region, except for one FN sample (**Figure 4**). For the modules we focused, Module 2 showed significantly differential CNV patterns between FP and FN-RMS (adjusted *p*-value 1.24E-50) (**Supplementary Table S5**). At transcriptomic level, Module 2 (adjusted *p*-value 2.56E-66), Module 18 (adjusted *p*-value 0.013198578), Module 34 (adjusted *p*-value 7.82E-10), and Module 41 (adjusted *p*-value 3.67E-07) presented differential expression patterns between FP-RMS and FN-RMS (**Supplementary Table S5**).

**Figure 5:**
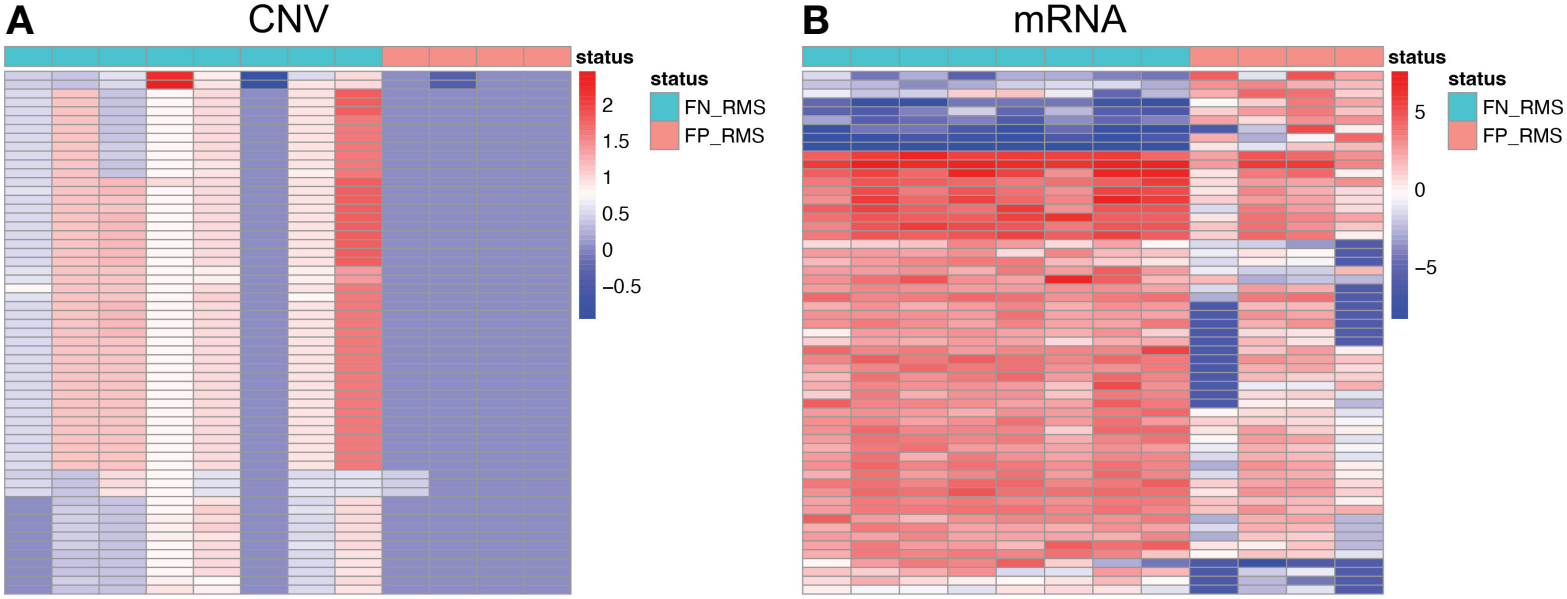
Heatmaps of Module 2 genes with consistent copy number and expression changes in FN-RMS vs FP-RMS in St. Jude dataset. **(A)** is for CNV; **(B)** is for mRNA.

For Module 2 genes, most of the expressions were elevated in the FN compared with FP samples (**Figure 5B**), which are consistent with the CN amplification in those regions in FN samples (**Figure 5A**). More specifically, 45.4% (229/504) of them display higher CN in FN-RMS *versus* FP-RMS samples. At the transcriptome level, 27.4% (138/504) of Module 2 genes are differential expressed between FN and FP-RMS cases and most of these DEGs were higher expressed in FN-RMS. Moreover, 59 of 229 of the genes discovered with CN alteration show significant changes on mRNA expression (**Figure 5**). Twenty-eight of 59 (47.5%) genes were also concentrated on the previously identified chr 8 cytobands (**Supplementary Table S6**). All these results indicated that Module 2 genes are highly altered at both genome and transcriptome level in FN samples, and the CN amplifications of chr 8 contribute significantly to the gene co-expression of Module 2, causing a large portion of the genes to be higher expressed (including *MYC*) and could further induce other downstream biological changes in FN-RMS.

Next, we further checked previously identified common regulators (i.e., *YAP1*, *TWIST1*, *KLF11*, *GATA4*, *MYC*, *MYCN*, *MYOG*, and *MYOD1*) in comparative analysis using St. Jude data, and five of eight TFs (*YAP1*, *MYC*, *TWIST1*, *GATA4*, and *MYOD1*) are significantly altered in both genomic or transcriptomic levels (**Table 4**). *YAP1* is located on chr11q22.1 and is the common upstream regulator of Module 18. It shows consistent higher CN and gene expression in FN-RMS *versus* FP-RMS cases in both discovery and St Jude datasets (**Table 4**). *MYC* is located on chr8q24.21, which is adjacent to the enriched cytobands we identified through co-expression, the same as *YAP1*, it presents consistent higher CN and mRNA expression (significantly amplified [*p*-value = 0.0174] in CN data and near significant upregulation expression data [*p*-value = 0.058]) in FN- *versus* FP-RMS cases of St. Jude data (**Table 4**). *TWIST1* is located on 7p21.1 with no CN change in St. Jude dataset but shows consistent higher expression in FN-RMS cases in both discovery and St Jude dataset (**Table 4**). *MYOD1* is located on 11p15.1. It exhibits lower gene expression in FN- *versus* FP-RMS cases in discovery datasets but shows CN amplification in St. Jude dataset with no significant gene expression change (**Table 4**). *GATA4* is located on chr8p23.1. It exhibits lower gene expression in FN- *versus* FP-RMS cases in discovery datasets but shows CN amplification in St Jude dataset with near significant downregulation (*p*-value = 0.057) in St. Jude expression dataset in FN- *versus* FP-RMS cases (**Table 4**). *KLF11* is located on 2p25.1. It exhibits no CN change in St. Jude dataset and near significant higher expression in FN-RMS cases, which is consistent with the changes of discovery datasets. As for the other two upstream regulators (*MYCN* and *MYOG*), no significant differences are observed between two groups at the both genome and transcriptome level in St. Jude dataset (**Table 4**), possibly due to the small sample size. We also checked *MYC*, *YAP1*, and *TWIST1* expression changes in FN-RMS with respect to normal tissue in GSE108022 and GSE28511. However, the two datasets showed inconsistent expression change for the three genes; therefore, their expression changes are inconclusive at this point. We expect more normal samples and larger cohort data in the future may give us more definitive answers on this aspect.

Taken together, these results confirmed that the CN amplification of chr 8 and the common regulators (*YAP1*, *MYC*, and *TWIST1)* work together to affect the downstream gene co-expression and contribute FN-RMS progression.

## 4 Discussion

RMS has been traditionally classified into embryonal RMS (ERMS) and alveolar RMS (ARMS) based on the histopathological features (1). Compared with histological classification, molecular classification based on the presence or absence of PAX3– or PAX7–FOXO1(PAX–FOXO1) fusion can more accurately capture the molecular biology patterns and cytological features of RMS and prognose patient outcome to guide clinical therapy (2), therefore has gained more and more popularity in the RMS research community in recent years. However, compared with the relatively clear fusion-driven oncogenic cascade events in FP-RMS, where the PAX3/7 gene fusion to FOXO1 creates a new fused transcriptional factor and completely altered the downstream targets’ expression profile, therefore serves as one of the driving mutations of FP-RMS, little is known about the molecular mechanisms for the tumorigenesis of FN-RMS which accounts for over 80% RMS cases. There is an urgent need to understand the disease etiology and develop new and effective therapeutic targets for this subtype.

In this study, we applied the fGCN analysis approach to explore the molecular mechanisms behind tumorigenesis in FN-RMS. fGCN analysis identified five consensus modules (Modules 2, 18, 34, 41, and 46), which present distinct expression patterns between FN *versus* FP RMS samples. Go enrichment analysis reveals that these modules were enriched in four major types of biological processes, that is, extracellular matrix organization, cell morphology, neuron development, and muscle structure processes. Davicioni et al. have demonstrated that neuronal and muscle structure related biological process were significantly altered between FP-RMS and FN-RMS (29), and our results confirmed that. As RMS is a skeletal muscle−derived soft tissue tumor, it is not surprising that muscle differentiation and its dysregulation are major contributors to FN-RMS tumorigenesis and progression, which are indicated by the differentially expressed fGCN modules 18 and 46. Cell morphology refers to the shape and size of cells and pathologists performed diagnosis and prognosis of disease based on the changes of cell morphology. In addition, the interaction of extracellular matrix (ECM) with tumor cells plays important roles in tumor invasion and metastasis. There is no doubt that the dysregulation of cell morphology and extracellular matrix related biological process promote FN-RMS development.

CN amplification of specifical cytobands has been observed in FN-RMS samples, although their roles in tumorigenesis were not further explored (15, 16, 30). Previous studies also discovered that ERMS samples (FN-RMS) displayed chromosomal region gains, including regions on chr 8 (30, 31). In our analysis, we observed the same CN amplifications on FN samples (**Figure 4**), and Module 2 genes were highly enriched on chr 8 with CN amplification and the consistent gene overexpression in FN-RMS samples (**Figure 5**) from our validation cohort. Fifty-nine genes from Module2 located on the identified chr 8 cytobands show CN amplification and elevated gene expression. They participate in biological processes such as protein transport and vesicle trafficking and are components of synapse, endoplasmic reticulum membrane, and Golgi apparatus. Not surprisingly, among them are *EGFR* (32, 33), *NCOA2* (34, 35), *DERL1* (36, 37), *EXT1* (38, 39), *PLAG1* (39), *COPS5* (40, 41), *ASAP1* (41, 42), *CHRNA6* (43, 44), and *CHRNB3* (44, 45), which have been associated with multiple types of cancers in previous studies. Therefore, the CN amplification not only explains the co-expression of this group of genes but also provides a potential driving force for FN-RMS and generates new potential therapeutic targets for this subtype.

We also discovered that multiple potential oncogenic transcriptional factors *MYC*, *YAP1*, and *TWIST1* with concordant CN and expression changes may contribute to FN-RMS tumorigenesis as well. *MYC*, which is located on chr8q24.21 and right next to the identified cytobands, showing consistent CN amplification and overexpression changes in FN-RMS samples (**Figure 3**) (**Table 4**). *MYC* is also the upstream regulator of Module 41 and 9.2–24% of the targets are differentially expressed compared FN-RMS with FP-RMS in the discovery datasets (**Table 5**). More importantly, as high as 45.8% of Myc target genes are differentially expressed in FN-RMS *versus* normal samples (**Table 5**). Accumulated evidences have suggested that *MYC* was an important oncogenic factor in RMS (46, 47), and its upregulation is closely related to tumor aggression and poor clinical outcome (46–51). Durbin et al. have highlighted that *MYC* could pathogenically subvert a myogenic core regulatory circuitry (CRC; the essential TFs of RMS) to promote RMS tumorigenesis and progression (52). They have also pointed that *MYC* was closely associated with the CN amplification (52). *MYC* higher expression were observed in ERMS (FN RMS) samples and FN-ARMS (53, 54). Moreover, several studies demonstrated that the depletion of *c-MYC* resulted the decrease of metastatic, invasive, and angiogenic-related markers (53, 55–57). Importantly, the inhibition of *MYC* was observed to decrease tumorigenicity of ERMS and myogenic differentiation (54). All these results agreed with our observation that the CN amplification of *MYC* contribute to the FN-RMS tumorigenesis and progression and *MYC* could serve as an attractive target for FN-RMS clinical treatment. *YAP1* also presents concordant CN amplification and overexpression in FN-RMS in both discovery datasets and St. Jude data (**Table 4**). This observation is consistent with another report showing CN amplification and overexpression of *YAP1* in ERMS (FN-RMS) compared with FP-ARMS (58). Meanwhile, we noticed that *YAP1* is the upstream regulator of Module 18, which presenting distinct expression patterns between FN- and FP-RMS are enriched with muscle differentiation and development genes, and about up to 27% of the YAP1/TEAD target genes are differential expressed in the FN- *versus* FP-RMS in the discovery datasets. Therefore, we proposed that *YAP1* CN amplification may also contribute to the different molecular patterns between two groups. Tremblay et al. found that reducing *YAP1* expression could decrease the expression of mature skeletal muscle differentiation related genes (58). Moreover, they have also highlighted that the hyperactivity of *YAP1* was highly related to the increased tumor stage and poor prognosis in ERMS and FN-ARMS (FN-RMS) and the knockdown of *YAP1* could reduce ERMS (FN-RMS) tumorigenicity. Although we did not observe consistent upregulation of *YAP1* in FN-RMS *versus* normal samples among different cohorts, we did observe that up to 43.1% *YAP1* target genes are significantly differentially expressed in this comparison. All these evidences suggest that *YAP1* is a potential oncogenic driver in FN-RMS. For another TF *TWIST1* we identified through fGCN mining, we observed consistent overexpression patterns in FN-RMS samples compared with FP-RMS at both discovery datasets and St. Jude dataset (**Table 4**). Maestro et al. have showed that *TWIST1* CN amplification and overexpression play important roles in RMS development (59). Furthermore, *TWIST1* play a key role in myogenesis and genetically or pharmacologically inhibition of *TWIST1* could curb cancer-driven muscle cachexia and reduce morbidity and mortality (60). Tremblay et al. have also discovered that *YAP1* could upregulate *TWIST1* to contribute ERMS (FN-RMS) development (58). This is consistent with our identification that both *YAP1* and *TWIST1* show higher expressed in FN-RMS. All these evidences support that *TWIST1* is another potential driver gene in RMS. *MYOD1* is a myogenic regulatory factor and is necessary for muscle fiber differentiation (61). However, its role in FN-RMS samples that we examined shows inconsistent results. In the discover datasets, we observed lower expression of *MYOD1* in FN-RMS samples compared with FP-RMS ones. While in the St. Jude dataset, *MYOD1* CN amplification is observed in FN-RMS, but no significant changes in term of gene expression between two fusion status subtypes. Zibat et al. identified that *MYOD1* is significantly lower expressed in FN-RMS (ERMS and FN-ARMS) than FN-RMS(PAX-FOXO1 ARMS) (62), and lacking of *MYOD1* is associated with poor prognosis in ERMS (61). In addition, *TWIST1* is a known *MYOD1* inhibitor (63) and Tremblay et al. have observed that *YAP1* and *TWIST1* could inhibit *MYOD1* activity in ERMS (58). This agrees with our finding that *YAP1*, *TWIST1*, and *MYOD1* showed inverse expression patterns (in the discovery dataset). As in the St. Jude dataset, we observed *MYOD1* CN amplification in FN-RMS than FP-RMS and no significantly expression difference, this may due to a relatively small sample size in this cohort and no normal skeletal muscles included for analysis. A large cohort with different fusion subtypes and normal skeletal muscles are needed for a definitive answer. For *GATA4*, it is right on the identified cytoband chr8p23.1 and shows downregulation in discovery datasets and St. Jude dataset transcriptomic level. It is involved in embryogenesis and in myocardial differentiation and function and is a common regulator for fusion status-associated Module 18 and 46. However, we observed CN amplification in St. Jude dataset FN *versus* FP RMS (**Table 4**). Therefore, *GATA4* role in term of tumorigenesis may require a large cohort data to verify.

In summary, all these findings demonstrate that the amplified CNs on chr8 genes and the overexpression of *MYC*, *YAP1*, and *TWIST1* may contribute to FN-RMS development, and they can serve as promising targets for FN-RMS therapeutic drug targets. Interestingly, our study also indicated that different molecular mechanisms in terms of genomics alteration exist among distinct RMS subtypes, which induce similar downstream transcriptomic changes and biological processes to manifest the similar tumor development in the skeletal muscle tissues.

Despite the extensive observations and consistent results generated from our analysis, some limitations of this study should be noticed as well. First, while the molecular mechanisms and the potential drivers have been largely defined, further experimental validation are still needed to confirm their function in FN-RMS. Second, as the two datasets contain very few normal skeletal muscles samples (five for GSE108022 and six for GSE28511), further larger scale comparative study between FN-RMS and normal skeletal muscles are still needed to verify the contribution of CN alteration and the three TF changes in FN-RMS tumorigenesis and progression. Third, even though the driving force of CNV has been revealed in FN-RMS, not all FN-RMS samples shown that CN changes. Other driving mechanism may also exist or co-exist in the FN-RMS patients. As FN-RMS is still a quite heterogeneous RMS subtype, the driving force can be multiple factors in multiple levels that work concordantly toward the tumorigenesis in different FN samples. It is very possible that they may possess other important somatic mutations and SNVs such as the ones in RAS pathway. As of now, we do not have the somatic mutation or SNV data for the samples that we investigated in our validation cohort. In our future work, when these data are available, we will separate the RAS pathway SNV and mutations from the wild-type samples, see how much CNV effects contribute to each subtype, and focus more on the RAS wild-type samples, which CNV may exert strong driving forces. Last but not the least, although *MYC*, *YAP1*, and *TWIST1* can be promising therapeutic targets, both pharmacologically and clinical trials assessment are still needed before clinical application. 

## 5 Conclusions

In conclusion, we identified the potential driver genes and investigate their molecular mechanism for PAX3/PAX7-FOXO1 fusion-negative RMS based on fGCN mining, which can also serve as promising therapeutical drug targets in FN-RMS. We confirmed that the CN amplification of specific cytobands (mostly on chromosome 8) and the elevated expression of the upstream regulators (such as *MYC*, *YAP1*, and *TWIST1*) work together to regulate the downstream gene differential expression and subsequent induce extracellular matrix organization, cell morphology, neuron development, and muscle structure–related biological processes dysfunction, which promoting FN-RMS tumorigenesis and development. Our findings not only helped to improve the understanding of the mechanisms of FN-RMS but also offered promising potential targets for FN-RMS personalized therapeutic intervention. Future works of manipulating these driver genes on FN-RMS cell lines are undergoing.

## Data availability statement

The datasets presented in this study can be found in online repositories. The names of the repository/repositories and accession number(s) can be found in the article/**Supplementary Material**.

## Author contributions

Conceptualization, JZ and KH; methodology, XZ, YL, AJ, and JZ; validation, XZ, AJ, SH, WW, and BY; formal analysis, YL; investigation, XZ; resources, PP and KP; data curation, XZ; writing—original draft preparation, XZ; writing—review and editing, XZ and JZ; visualization, XZ; supervision, JZ, KH, JR, and XY; project administration, JZ, KH, and JR; funding acquisition, XZ, JZ, KH, JR, and XY. All authors contributed to the article and approved the submitted version.

## Supplementary material

Supplementary Table 1 The 50 consensus modules. xlsx

Supplementary Table 2 Modules associated with fusion status in discovery datasets. xlsx

Supplementary Table 3 The enriched biological processes and cytobands of the 50 fGCN modules. xlsx

Supplementary Table 4 Cytoband enrichment for Module 2 DEGs between FN-RMS and normal tissue.

Supplementary Table 5 The molecular patterns of the five fusion status associated modules in St. Jude dataset. xlsx.

Supplementary Table 6 Chromosome location of St. Jude Module 2 genes with changes in both CNV level and mRNA level. xlsx.

Supplementary Figure 1 Heatmap of Module 2 differential-expressed genes with respect to normal tissue.
