# Supplementary figures and images for: Identify potential driver genes for PAX-FOXO1 fusion-negative rhabdomyosarcoma through frequent gene co-expression network mining
Source: Front Oncol. 2023 Jan 30;13:1080989. doi: 10.3389/fonc.2023.1080989 (PMC9924292; doi:10.3389/fonc.2023.1080989)

A

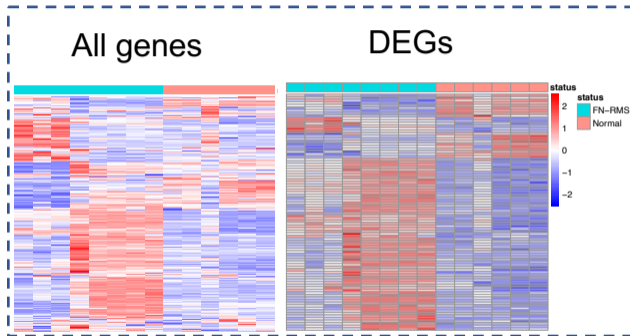

GSE28511

Module2

B

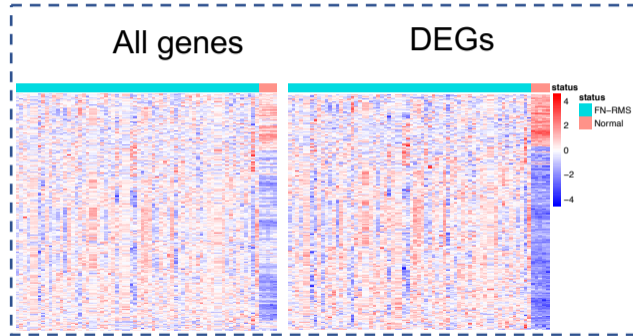

GSE108022

Supplement: Supplementary Figure 1 — Heatmap of Module 2 differential-expressed genes with respect to normal tissue. [file DataSheet_1.pdf]
